# Medicinal Ingredients of Wax Gourd (*Benincasa hispida* (Thunb.) Cogn.): An Integrated Review of Phytochemistry, Pharmacology, and Nutraceutical Applications

**DOI:** 10.3390/plants15132020

**Published:** 2026-06-30

**Authors:** Qiancheng Mao, Xuling Zhai, Jinqiang Yan, Wenrui Liu, Piaoyun Sun, Qian Zhou, Biao Jiang

**Affiliations:** 1 Vegetable Research Institute, Guangdong Academy of Agricultural Sciences, Guangzhou 510640, China; 15588328849@163.com (Q.M.);; 2 Guangdong Key Laboratory for New Technology Research of Vegetables, Guangzhou 510640, China; 3 College of Agriculture and Biotechnology, Sun Yat-sen University, Shenzhen 518107, China

**Keywords:** wax gourd, *Benincasa hispida*, phytochemistry, pharmacology, bioactive compounds, nutraceuticals, traditional medicine

## Abstract

Wax gourd (*Benincasa hispida* (Thunb.) Cogn.), a monotypic member of the *Cucurbitaceae* family, is a globally significant vegetable crop distinguished by its versatile nutritional and therapeutic properties. This review systematically synthesizes current knowledge on the phytochemical composition and pharmacological potential of various botanical parts, thereby bridging ethnobotanical history with modern molecular insights. Recent advancements in multi-omics technologies, particularly metabolomics and chromatography–mass spectrometry, have significantly deepened the characterization of its bioactive constituents, including specific proteins, enzymes, peptides, flavonoids, triterpenes, sterols, and polysaccharides. Special emphasis is placed on the molecular mechanisms and biosynthetic pathways underlying its pharmacological activities-ranging from antioxidant, anti-inflammatory, and immunomodulatory effects to metabolic regulation involving anti-ulcer, nephroprotective, and hypotensive properties. Recent investigations have identified over 60 bioactive compounds, including cucurbitacins, flavonoids, polysaccharides, and phenolic acids in wax gourd, and elucidated their anti-diabetic, anti-cancer, and organ-protective effects through multiple signaling pathways. By integrating traditional medicinal applications with emerging genomic insights-including recent whole-genome sequencing and QTL mapping studies-this review highlights the current understanding of the genetic basis underlying fruit quality and discusses the potential for molecular breeding approaches and the development of functional nutraceuticals. Ultimately, wax gourd represents a valuable candidate for leveraging horticultural science to enhance human health and disease management.

## 1. Introduction

Wax gourd (*Benincasa hispida* (Thunb.) Cogn.), the sole species of genus Benincasa native to Southeast Asia and Oceania, with China and Eastern India as core domestication and cultivation centers. It also holds a prominent place in traditional medicine systems, including Ayurveda and Traditional Chinese Medicine [1]. It is now widely distributed and cultivated throughout the tropical and subtropical regions of Asia. While extensively utilized as a primary food source, this plant also holds a prominent place in ethnomedicine. The growing global interest in plant-derived nutraceuticals and functional foods has renewed attention to underexploited vegetable crops with documented ethnobotanical significance.

This review aims to synthesize the accumulating body of research on wax gourd, with particular emphasis on its phytochemistry and pharmacology, thereby addressing a gap in the current literature. To accomplish this objective, a comprehensive literature search was performed using multiple databases, including PubMed, ScienceDirect, SpringerLink, and Google Scholar. The research strategy incorporated several targeted keywords, such as “biological activities”, “pharmacological effects”, “phytochemical composition”, “traditional medicinal applications”, “toxicological profile”, and “safety evaluation”. Peer-reviewed publications providing experimental evidence on molecular mechanisms underlying traditional applications were prioritized. Taxonomic verification was cross-referenced using “The Plant List” and the PubChem database. Additional sources including specialized books and technical reports were consulted to ensure adequate coverage of its bioactive properties.

The pharmacological potential of wax gourd arises from its diverse profile of bioactive compounds, which include phenolic acids, flavonoid derivatives, triterpenoids, proteins and enzymes. Wax gourd has a cultivation history of more than 2000 years, with an annual output of more than 15 million tons in China alone, making it one of the top five vegetable crops. It has been used in traditional medicine to treat obesity, ulcers, epilepsy, urinary system diseases, inflammation and metabolic disorders [2,3]. By integrating ethnomedicinal documentation with modern experimental findings on its nutritional and pharmacological applications, this review offers a critical evaluation of the medicinal properties of wax gourd.

In this context, wax gourd stands out as a species where traditional medicinal knowledge converges with modern pharmacological evidence, warranting a systematic synthesis of available data. While previous reviews have addressed the phytochemistry and pharmacology of wax gourd, the present review distinguishes itself by: (1) integrating recent multi-omics findings, including whole-genome sequencing and metabolomic profiling; (2) providing a critical assessment of the translational gaps between preclinical evidence and clinical applications; (3) systematically evaluating the nutraceutical potential with consideration of bioavailability, pharmacokinetics, and formulation challenges; and (4) proposing a future research agenda centered on molecular breeding and evidence-based functional food development.

## 2. Botanical Characteristics

Wax gourd is a robust, annual trailing or climbing herb. The stems are thick, stout, angular, and furrowed, covered with hispid hairs and bearing branched tendrils. The leaves are hispid and triangular, measuring up to 25 cm in both length and width. As a monoecious species, it exhibits sexual dimorphism with solitary male and female flowers appearing on the same plant. The flowers are yellow with rotate-campanulate corollas. Male flowers are 5–15 cm in length and contain three stamens approximately 1 cm long, while female flowers feature an inferior ovary (2–4 cm long) with a trilobed style [4].

The fruits exhibit striking phenotypic diversity in size and shape, ranging from spherical to oblong, and are among the largest in the Cucurbitaceae family-reaching lengths of up to 1 m and weights exceeding 40 kg. In the early stages of development, the fruit is green and covered with fine hairs. As it matures, it develops a hard pericarp (rind) overlain with a characteristic chalk-white waxy bloom, which significantly enhances its postharvest shelf life. The inner mesocarp (flesh) is white, spongy, and succulent. The seeds are ovate, compressed (flat), and typically measure 1 cm in length [2].

### 2.1. Traditional Applications

In traditional Ayurvedic medicine, different parts of wax gourd, including leaves, fruits, seeds, and roots are used in medicinal formulations to balance bodily humors, especially pitta (metabolic/thermoregulatory) and kapha (structural/mucosal) disorders [3]. Wax gourd exhibits extensive pharmacological activities affecting the respiratory, neurological, gastrointestinal, and metabolic systems, and is traditionally applied as a detoxifying agent, libido booster and digestive stimulant. Its clinical and traditional uses cover respiratory distress, convulsive disorders, epilepsy, bronchial diseases, diaphragmatic spasms, hemoptysis and urinary retention.

In the Indian subcontinent, the fleshy mesocarp is often made into Petha Cubes, a traditional candied sweet widely used in vegetarian diets. Representative preparations such as Kushmanda Ghrita(KG) and Lehyam serve as nutritional supplements, diuretics, and tonics, and are effective in managing neurological disorders including seizures, urinary abnormalities and gastrointestinal diseases [5].

In Traditional Chinese Medicine, the dried exocarpium of wax gourd (known as *Dongguapi* or *Benincasae Exocarpium*) has been officially recorded in the Chinese Pharmacopeia for its diuretic, anti-edematous, and heat-clearing properties. A review by Zhang et al. [6] has systematically summarized the ethnopharmacology, phytochemistry, and pharmacology of *Benincasae Exocarpium*, identifying over 60 chemical constituents and documenting its diuretic, nephroprotective, and anti-inflammatory activities, thereby providing a valuable complementary perspective to the whole-fruit focus of the present review.

### 2.2. Global Distribution

The widespread traditional use of wax gourd across disparate cultures is reflected in its broad geographical distribution and domestication history, which together provide the biogeographic context for the plant’s ethnopharmacological significance.

According to Kew Science’s Plants of the World Online database, wax gourd is native to the Bismarck Archipelago, Borneo, Jawa, the Lesser Sunda Islands, Maluku, New Caledonia, New Guinea, Queensland, Solomon Islands, and Sulawesi [7]. Java has been proposed as one of the domestication centers of wax gourd, although the exact origin remains debated, with other regions including Japan and Indo-China also suggested as potential centers of diversity. The species has been widely introduced and cultivated across tropical and subtropical regions of Asia, including China, India, Bangladesh, Sri Lanka, and Southeast Asian nations, where it holds major agricultural and medicinal importance [1,8].

In East Asia, wax gourd has been cultivated for over 2000 years, with China as the primary center of modern cultivation and domestication and the largest global producer [8]. India represents the second major production region, where the crop is integral to both agricultural systems and Ayurvedic traditional medicine [3]. In Southeast Asia, wax gourd is widely grown in Vietnam, Thailand, the Philippines, and Malaysia, with diverse landraces adapted to local agro-climatic conditions [1]. The species has also been introduced to Africa, the Caribbean, and South America, although commercial production in these regions remains limited [7].

**Figure 1:**
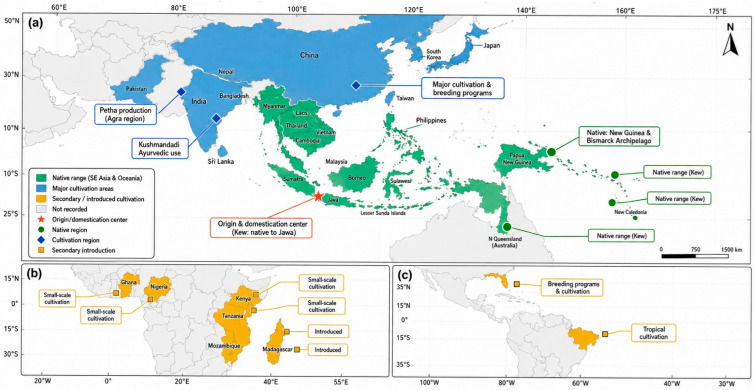
Global distribution of native habitats and cultivation regions of wax gourd (*Benincasa hispida*). (**a**) Native range in Southeast Asia and Oceania, the domestication center in Java (Indonesia), core cultivation and breeding areas in East and South Asia, as well as traditional medicinal and commercial planting regions in the Indian subcontinent; (**b**) Small-scale introduced cultivation areas across West, East Africa and Madagascar; (**c**) Introduced tropical cultivation and regional breeding bases in the Americas and Caribbean. Green, blue, and orange colors denote native range, major cultivation zones, and secondary introduced cultivation areas, respectively. The red star indicates the domestication origin; green circles represent native distribution records; blue diamonds mark key cultivation and breeding regions. The scale bar in panel (**a**) corresponds to a maximum distance of 1500 km.

The global distribution of wax gourd reflects both its ancient dispersal through natural mechanisms and its more recent spread through human-mediated cultivation. Phylogeographic evidence suggests that the species originated in the Indo-Malayan region before spreading northward into China and westward into the Indian subcontinent [7,8]. A distribution map illustrating the native range and major cultivation areas is presented in Figure 1, highlighting the pan-tropical distribution of this economically important cucurbit. The native range encompasses the Indo-Malayan region (green), while major cultivation zones (blue) extend across East, South, and Southeast Asia, with secondary introductions (orange) in Africa and the Americas.

Recent comparative chloroplast genome analysis by Song et al. [9] clarified the phylogenetic relationships among cucurbit species and confirmed that wax gourd forms a monotypic lineage within the tribe *Benincaseae*. These genomic data complement the morphological and biogeographic evidence for the species’ origin and dispersal history.

## 3. Overview of Bioactive Compounds

### 3.1. Nutritional Composition

Phytochemical and nutritional analyses of wax gourd reveal that the fruit is rich in essential oils, flavonoids, carbohydrates, proteins, glycosidic derivatives, carotenoids, vitamins, uronic acid derivatives, and *β*-sitosterol. Its peel primarily contains galactose, glucose, xylose, and sorbose [10,11].

Seeds are also nutritionally notable, containing approximately 11.6% protein, and 20.7% lipids (Table 1). Seed oil is dominated by linoleic acid (67.4%), followed by palmitic acid (17.1%), oleic acid (8.4–10.2%), and stearic acid (4.8%) [12,13]. Whole-plant phytochemical profiling further revealed detectable glycosides and alkaloids, consistent presence of tannins (assessed via chromogenic reaction intensity and precipitation characteristics), while free carbohydrates and lipids were undetectable in these specific extracts under the employed analytical protocols [10,14].

The content of active substances varies significantly among different tissues: the peel has the highest cucurbitacin content (2.34 mg/g DW, dry weight) and total flavonoids (35–45 mg/100 g DW), followed by leaves (1.85 mg/g DW cucurbitacins, 25–35 mg/100 g DW flavonoids), and the flesh is rich in polysaccharides (50–200 mg/g DW) [15,16]. Detailed phytochemical profiles of different tissues of wax gourd, including roots, fruits, seeds, leaves and stems, are comprehensively summarized in Supplementary Table S1.

### 3.2. Comprehensive Nutritional Profile of Wax Gourd Fruit

Table 1 presents the nutritional profile of wax gourd fruit, integrating data from multiple sources to provide a complete overview of macronutrients, micronutrients, vitamins, and amino acids [17]. It features a high water content of 96.1% and only 13 kcal per serving, along with 0.40 g protein, 0.1 g lipids, 1.9 g carbohydrates, 1 mg ascorbic acid, 0.80 g dietary fiber, 0.39 g ash, 0.8 mg iron and 30 mg calcium; mature fruits are rich in thiamine (B1), ascorbic acid I and dietary fiber, which makes up nearly 27.5% of its dry weight, and also contains high levels of organically bound amino acids, essential minerals and nucleoside derivatives that contribute to its antioxidant and anti-inflammatory effects, with potassium being the most abundant mineral [18]. All values are expressed per 100 g edible portion unless otherwise specified [3,17,18].

### 3.3. Proteins, Enzymes, and Peptides

#### 3.3.1. Ribosome-Inactivating Proteins, Proteases, and Enzyme Inhibitors

The wax gourd harbors multiple bioactive proteins, including hispin-a ribosome-inactivating protein with potent antifungal and antibacterial activity [19]. Scientific studies have successfully purified proteases like cucumisin and serine-type proteinases from the fruit pulp, demonstrating peak enzymatic efficiency at pH 8.6 and 60 °C while showing selective affinity for particular substrates [3]. In vitro studies have revealed the cytotoxic potential of these proteins. The identification of cucumisin-like proteolytic enzymes in the fruit enhances the fruit’s antimicrobial properties and suggests potential immunoregulatory applications [3]. Bioactive enzyme extracts derived from different plant components exhibit dual functionality, demonstrating both anticancer properties and significant involvement in modulating inflammatory pathways [20].

*Hispin* isolated from wax gourd further expands this library of bioactive molecules and opens avenues for biotechnological use in crop protection and therapeutic development. Ribosome-inactivating proteins (RIPs) form a large family of plant defensive proteins with conserved functional mechanisms: they trigger N-glycosidase-mediated depurination of a conserved adenine site within 28S rRNA, thereby blocking overall protein synthesis [21]. Members of the RIP family have been isolated from numerous genera of the Cucurbitaceae, and they display broad-spectrum antiviral, antifungal, and antitumor bioactivities. The characterization of hispin further expands the known repertoire of RIPs within cucurbit crops.

#### 3.3.2. Notable Peptides (α- and β-Benincasins)

Wax gourd seeds also synthesize ribosome-inactivating proteins including *hispin*, as well as *α*- and *β*-benincasins—arginine/glutamine-rich peptides with strong translational inhibitory and antifungal properties. Seed tissue represents a rich reservoir of these bioactive polypeptides, predominantly *α*- and *β*-benincasins distinguished by high arginine and glutamine contents [19]. These compounds suppress ribosomal translation, and ongoing preclinical research explores their therapeutic capacity to disrupt signaling cascades driving tumor proliferation and microbial infection [22].

### 3.4. Flavonoids and Phenolic Compounds

Recent phytochemical investigations have revealed an extensive array of bioactive compounds in wax gourd. Different plant parts including fruits, foliage, seeds, and root systems contain various chemical constituents spanning antioxidants, protein complexes, peptide derivatives, flavonoid compounds, triterpene structures, steroidal components, and carbohydrate polymers [13,23]. This comprehensive chemical composition underpins the plant’s pharmacological uses across various traditional and modern medical practices.

Wax gourd contains abundant phenolic acids such as gallic acid alongside various flavonoids such as catechin, naringenin, and astilbine, which exhibit strong antioxidant properties, anti-inflammatory effects, and gastric ulcer prevention capabilities [24,25]. Advanced detection methods including high-performance liquid chromatography and nuclear magnetic resonance spectroscopy have verified the existence of these phytochemicals [10,13]. These bioactive substances play a key role in neutralizing free radicals (particularly superoxide anions and hydroxyl radicals), thereby facilitating crucial biological mechanisms ranging from cytoprotection to regulation of inflammatory responses [25,26].

Comprehensive phytochemical profiling demonstrates elevated phenolic compound concentrations coupled with strong radical scavenging capacities in both epicarp and seed extracts, highlighting their essential contribution to the plant’s therapeutic applications [13,27].

The phytochemical profile contains diverse bioactive compounds including flavonoids, anthocyanins, terpenoids, saponins, tannins, steroids, carotenoids, glycosides, and resins, with the composition primarily featuring cucurbitacins, prominent triterpenoid compounds [15,23]. Analytical studies on 100 g edible portions of wax gourd seeds revealed considerable phenolic content and antioxidant capacity. Seed oil analysis demonstrated total tocochromanol concentrations ranging from 961.8 to 1027.6 µg/g, comprising *α*-tocopherol (2–3%), *β*-tocopherol (46.6–61.7%), *γ*-tocopherol (23–24.9%), *γ*-tocotrienol (6.8–8.2%), and *δ*-tocopherol (4.6–19.2%) [10,13]. Chromatographic analysis identified multiple phenolic acids including gallic acid, protocatechuic acid, 3,4-dihydroxybenzaldehyde, vanillic acid, vanillin, para-coumaric acid, trans-cinnamic acid, and ferulic acid, with concentrations varying between 12.27 and 17.25 µg/g in the extracted oils [10].

The bioavailability of flavonoids from wax gourd remains poorly characterized. Chen et al. [28] have comprehensively reviewed the absorption, metabolism, and bioavailability of dietary flavonoids, highlighting that factors such as glycosylation pattern, food matrix effects, and gut microbiota-mediated biotransformation significantly influence their systemic bioavailability. The flavonoids identified in wax gourd, including O-glycosylated and C-glycosylated derivatives, likely follow similar metabolic pathways, though specific pharmacokinetic studies on wax gourd flavonoids are lacking and represent an important research gap.

### 3.5. Triterpenes, Sterols, and Fatty Acids

The wax gourd contains a diverse array of triterpenes, including bryonolic acid and 7-oxodihydrokarounidiol-3-benzoate, along with sterols such as *β*-sitosterol and distinctive compounds including 24*β*-ethylidene cholesterol-7-enol derived from its seeds [13,29]. Oils obtained through ultrasound-assisted extraction methods have demonstrated elevated levels of health-promoting fatty acids, highlighting the plant’s potential as a functional dietary component [10,13].

Among the triterpenoids, cucurbitacins are of particular pharmacological interest due to their potent biological activities. Comprehensive reviews by Varela et al. [30] and Li et al. [31] have detailed the molecular mechanisms of cucurbitacin-mediated anticancer effects, including inhibition of the JAK/STAT3 signaling pathway, disruption of PI3K/Akt/mTOR signaling, induction of autophagy and apoptosis, and suppression of epithelial–mesenchymal transition (EMT). While these mechanisms have been primarily characterized for cucurbitacins from other Cucurbitaceae species (e.g., *Cucurbita*, *Momordica*), the structural conservation across the family suggests analogous pharmacological potential for those present in wax gourd, warranting targeted investigation.

Structure-activity relationship (SAR) analyses of cucurbitacins have revealed that the *α*,*β*-unsaturated ketone moiety in the side chain is critical for their biological activity, as it serves as a Michael acceptor for nucleophilic addition by cysteine residues in target proteins such as STAT3 and tubulin [30,31]. The acetylation pattern of hydroxyl groups at C-2, C-3, and C-24 further modulates the potency and selectivity of individual cucurbitacin compounds. For instance, cucurbitacin B and D, which bear free hydroxyl groups, exhibit distinct anti-inflammatory and anticancer profiles compared to their acetylated counterparts. Understanding these SAR principles is essential for the rational design of cucurbitacin-derived therapeutics with improved efficacy and reduced toxicity. Notably, cucurbitacins exhibit dose-dependent cytotoxicity, necessitating careful dosage control in therapeutic applications.

The above SAR rules are summarized from cucurbitacins of Momordica and Cucurbita; structure-activity experiments specifically targeting wax gourd cucurbitacin B and D remain blank, which is a key direction for subsequent pharmacological research.

### 3.6. Polysaccharides and Other Minor Constituents

The cell walls are rich in polysaccharides like pectic compounds, which play a dual role in maintaining cellular architecture and modulating immune responses [5,32]. These complex biomolecules synergize with essential micronutrients including vitamins and mineral elements to enrich the dietary profile of wax gourd, reinforcing its application in therapeutic nutrition strategies [5,23].

The immunomodulatory activity of wax gourd polysaccharides (WGP) is thought to be mediated primarily through two interconnected pathways. The first involves the Toll-like receptor 4 (TLR4)/nuclear factor kappa-B (NF-κB) signaling axis. Plant-derived polysaccharides can be recognized by pattern recognition receptors on immune cells, particularly TLR4 on macrophages, triggering downstream MyD88-dependent signaling that culminates in NF-κB activation and the subsequent release of pro-inflammatory cytokines and nitric oxide [33]. This mechanism has been extensively characterized for polysaccharides from other Cucurbitaceae members and is likely conserved in WGP. In wax gourd, pectins constitute the dominant polysaccharide fraction. The second pathway involves gut microbiota-dependent modulation, where dietary polysaccharides serve as prebiotic substrates that promote the growth of beneficial commensal bacteria. These bacteria produce short-chain fatty acids, which exert systemic immunoregulatory effects. Together, these complementary mechanisms underpin the immunomodulatory potential of wax gourd polysaccharides.

The extraction methodology significantly influences the yield, molecular weight distribution, and biological activity of wax gourd polysaccharides. Conventional hot water extraction typically yields polysaccharides with molecular weights ranging from 10 to 500 kDa, whereas ultrasound-assisted extraction and enzymatic methods can produce fractions with distinct structural features and enhanced immunomodulatory potency [12]. Wang et al. [12] demonstrated that wax gourd polysaccharides extracted under optimized conditions exhibited notable moisture-retention and antioxidant properties, suggesting their dual utility in both cosmetic and pharmaceutical applications. Hot water extraction obtains high-molecular-weight WGP (100–500 kDa) with strong immunomodulatory activity; ultrasonic and enzymatic hydrolysis degrade polysaccharide chains to low-molecular fractions (10–80 kDa), which exhibit better moisture retention and antioxidant activity for cosmetic use [12]. The relationship between polysaccharide fine structure-particularly degree of branching, monosaccharide composition, and glycosidic linkage patterns-and specific immunomodulatory outcomes remains an active area of investigation in the broader plant polysaccharide field [33]. The chemical structures of key small-molecule bioactive constituents discussed in Section 3.3, Section 3.4, Section 3.5 and Section 3.6 are illustrated in Supplementary Figure S1, which includes representative phenolic compounds, flavonoids, tocopherols, triterpenoids, and sterols isolated from different tissues of wax gourd. These structurally diverse compounds collectively underpin the broad-spectrum pharmacological activities of wax gourd described in subsequent sections.

## 4. Pharmacological Activities

### 4.1. Antioxidant and Free Radical Scavenging Effects

Studies have consistently demonstrated wax gourd’s potent antioxidative properties, primarily attributable to its high phenolic compound and flavonoid content. Research findings from both laboratory experiments and animal models reveal that plant-derived extracts exhibit significant free radical neutralization capabilities. These extracts effectively counteract oxidative stress by inhibiting lipid peroxidation and preventing cellular membrane damage, while simultaneously enhancing the activity of key antioxidant enzymes such as superoxide dismutase and catalase [34].

The antioxidant defense of wax gourd derives from its complex phytochemistry, in which phenolic acids and flavonoids directly neutralize reactive oxygen species [14,35]. Gastric acid suppression in ulcer prevention potentially involves histamine receptor antagonism and neural pathway modulation. Bioactive proteins and peptides may interface with cellular translational processes, modulating programmed cell death pathways while activating immune cell signaling [25]. Vascular relaxation is attributed to nitric oxide-mediated signaling, whereas immune regulation is primarily driven by cytokine modulation and lymphocyte activation [22,36]. These antioxidant mechanisms intersect with other pharmacological pathways discussed in subsequent sections.

The antioxidant defense system of wax gourd operates through multiple complementary mechanisms. The phenolic hydroxyl groups of flavonoids such as catechin and quercetin derivatives directly neutralize reactive oxygen species (ROS) through hydrogen atom transfer (HAT) and single electron transfer (SET) mechanisms. Additionally, these compounds can chelate transition metal ions (Fe^2+^, Cu^2+^) that catalyze Fenton reactions, thereby preventing the generation of highly reactive hydroxyl radicals. At the cellular level, certain wax gourdH:quinone oxidoreductase 1 (NQO1), and glutathione S-transferases (GSTs) [3]. This dual mechanism-direct radical scavenging combined with induction of endogenous antioxidant defenses-underlies the potent cytoprotective effects observed in both in vitro and in vivo models. constituents activate the nuclear factor erythroid 2-related factor 2 (*NRF2*)/Kelch-like ECH-associated protein 1 (*KEAP1*) antioxidant response pathway, upregulating endogenous defense enzymes including heme oxygenase-1 (HO-1), NAD(P).

Mandana et al. [10,37] investigated the antioxidative properties of wax gourd seed extracts through DPPH (515 nm) and ABTS (734 nm) radical neutralization assays. Comparative analysis revealed that the ethanol-based extract displayed the strongest radical-scavenging capacity, outperforming ethyl acetate and *n*-hexane derivatives. Researchers attributed this enhanced bioactivity to the abundance of linoleic acid constituents identified in the ethanolic fraction.

Samad et al. [11] conducted a study on the antioxidant properties of aqueous extracts from dried wax gourd seeds. The total phenolic content (TPC) and flavonoid levels were quantified as 81.3 ± 1.4 μg gallic acid/g and 486.8 ± 4.1 μg catechin/g dry weight, respectively. Experimental analyses revealed inhibitory effects of 79.8% ± 0.2% in DPPH radical scavenging, 82.3% ± 1.9% in ABTS radical neutralization, and 95.5% ± 0.8% in hydroxyl radical elimination assays. Maximum linoleic acid inhibition reached 73.2% ± 1.0% following six-day incubation, while nitrite scavenging activity demonstrated 73.6% ± 1.0% efficacy within one hour of treatment.

### 4.2. Anti-Inflammatory and Immunomodulatory Properties

Wax gourd extracts exert anti-inflammatory effects primarily through suppression of cyclooxygenase (COX) pathways and reduced production of prostaglandins and thromboxanes. These preparations regulate cytokine synthesis and reduce inflammatory biomarkers, thereby alleviating inflammation-induced tissue injury [3,13]. These anti-inflammatory effects are accompanied by analgesic activity operating through both peripheral and central mechanisms, including putative opioid receptor interaction and suppression of histamine release [38]. Additionally, wax gourd exhibits immunoregulatory activity, modulating immune cell function to enhance host defense.

Recent studies have further elucidated the molecular mechanisms underlying the anti-inflammatory effects of wax gourd. IIn vitro studies demonstrated that ethanolic extracts of wax gourd fruit significantly suppressed the expression of pro-inflammatory cytokines, including tumor necrosis factor-alpha (TNF-α), interleukin-1 beta (IL-1β), and interleukin-6 (IL-6), in lipopolysaccharide (LPS)-stimulated macrophages [3,13,39]. The suppression of these cytokines was associated with inhibition of the nuclear factor kappa B (NF-κB) signaling pathway, a master regulator of inflammatory gene transcription. 

The flavonoid constituents of wax gourd, particularly rutin aId isovitexin, are identified as key contributors to its immunomodulatory activity. Rutin inhibits cyclooxygenase-2 (COX-2) expression and prostaglandin E2 (PGE2) production through modulation of the MAPK/ERK signaling cascade [38]. Isovitexin exhibited significant inhibition of nitric oxide (NO) production in LPS-activated RAW 264.7 macrophage cells, with an IC50 value indicating potent anti-inflammatory capacity [40]. Furthermore, the triterpenoid component bryonolic acid, isolated from wax gourd roots, was found to suppress inducible nitric oxide synthase (iNOS) expression through interference with AP-1 transcription factor activation [16].

In vivo anti-inflammatory assessments using carrageenan-induced rat paw edema models revealed that oral administration of wax gourd aqueous extract at doses of 200–400 mg/kg produced a significant reduction in edema volume, comparable to the standard drug indomethacin [3,38]. The anti-inflammatory response was dose-dependent and sustained over the 5 h observation period. Additionally, the immunostimulatory wax gourd mitogen (BSM), a heteropolysaccharide isolated from seed extracts, demonstrated the capacity to activate peritoneal macrophages and enhance splenocyte proliferation in murine models, indicating dual anti-inflammatory and immunomodulatory potential [26]. These findings collectively support the therapeutic relevance of wax gourd phytochemicals in managing inflammatory and immune-mediated conditions.

### 4.3. Gastroprotective and Anti-Ulcerogenic Activity

Wax gourd demonstrates well-documented anti-ulcer activity in gastrointestinal disorders. Preclinical studies show that fresh juice and various preparations effectively manage chemically induced gastric lesions through multiple mechanisms. These formulations suppress gastric acid production, enhance mucosal protection, and promote tissue regeneration via histaminergic pathway inhibition and cholinergic regulation. Hydroalcoholic and methanol fractions reduce ulcer severity in animal models primarily through enhanced antioxidant activity and preserved gastric epithelial integrity [23].

Kumazawa et al. [26] conducted the isolation and analysis of immunostimulatory properties in the wax gourd mitogen (BSM) fraction derived from wax gourd seed extracts seed extracts. The BSM component was identified as a heteropolysaccharide containing uronic acid residues, neutral carbohydrates, proteinaceous elements, and phosphate groups. Immunomodulatory effects manifested through BSM-induced proliferation of murine B lymphocytes and differentiation into antibody-producing cells, coupled with macrophage activation into tumoricidal variants during in vitro experiments. Furthermore, this compound enhanced IgM/IgG antibody production against sheep red blood cell antigens and amplified delayed-type hypersensitivity responses in murine models. Researchers hypothesized that the observed bioactivities may be linked to the presence of bacterial lipopolysaccharide contaminants.

Grover et al. [23] demonstrated that various preparations of wax gourd-including fresh juice, supernatant, residue, methanolic extract, and petroleum ether extract-exhibited inhibitory effects on experimental ulcer formation. Their study employed ranitidine (100 mg/kg oral administration) as a reference treatment, administered thirty minutes before ulcerogen exposure. Distinct biological responses emerged among the different fruit-derived preparations. In swimming stress-induced ulcer models, fresh juice administration at 4 mL per experimental subject demonstrated statistically significant anti-ulcer properties. Comparative analysis revealed that fresh juice doses of 2 mL per subject achieved significant efficacy (significant) in mitigating ulcer dimensions across aspirin-restraint and serotonin-induced ulcer models. Experimental data further indicated that wax gourd fresh juice, supernatant, and alcohol-based extract collectively reduced ulcer severity in swimming stress models. Researchers attributed these therapeutic outcomes to wax gourd’s capacity to modulate gastric acid secretion through pharmacological antagonism.

Qadrie’s yeast-induced fever models, showing 28.6% and 39.2% temperature reduction at respective doses that showed equivalence to the reference medication aspirin (150 mg/kg). These pharmacological outcomes validate the ethnomedicinal application of wax gourd for pain relief and fever management. Qadrie et al. [32] demonstrated time-dependent and dose-responsive elevation of mechanical pain thresholds following administration of wax gourd seed ethanol extract. Research revealed statistically significant enhancement of antinociceptive effects particularly at 250 and 500 mg/kg doses, with flavonoid content identified as the probable bioactive component. Concurrently, the extract exhibited dose-responsive antipyretic activity in 15% Brewer.

Wax gourd demonstrates significant anti-ulcer efficacy through multi-mechanistic actions. Preclinical studies reveal that its extracts (fresh juice, alcoholic, and petroleum ether fractions) effectively reduce ulcer indices in diverse models, including stress-induced and drug-mediated ulcers. The activity is particularly dose-dependent in stress-related paradigms, suggesting central nervous system involvement. Additional mechanisms encompass antihistaminic effects, anticholinergic activity, and preservation of gastric microcirculation. Chronic toxicity assessments confirm the safety of its fresh juice, showing no hematological or biochemical alterations. These findings validate its traditional use and position wax gourd as a multi-targeted natural therapeutic for ulcer prevention [23].

Sastry et al. investigated the effects of wax gourd extracts on gastric acid secretion in rodent models. Their research revealed that the aqueous extract demonstrated notable protective capabilities regarding gastric pH stabilization and gastric juice volume regulation statistically significant [39]. This extract also exhibited corrective effects on both free and total acidity parameters while significantly preserving vitamin C concentrations in gastric secretions, effects attributed to its secretagogue properties. Concurrently, the ethyl acetate extract displayed dual functionality as both an antioxidant agent and inhibitor of lipid peroxidation processes.Sastry et al. investigated the therapeutic potential of various solvent extracts from wax gourd in addressing experimental.

### 4.4. Cytotoxic, Antiviral, and Larvicidal Effects

Bioactive properties of wax gourd have been validated through laboratory-based toxicity assessments using brine shrimp models. Methanol-based seed derivatives demonstrated cytotoxic effects with low median lethal concentrations, indicating possible therapeutic or pesticidal applications. Research further reveals that water-soluble fractions contain macromolecular components with inhibitory effects against Columbia S. K. viral strains [41]. Botanical investigations additionally highlight the plant’s insecticidal capacity, where foliar extracts exhibited substantial efficacy against culicidae larvae populations-findings with potential applications in vector control [40].

At the molecular level, the anti-cancer properties of wax gourd are increasingly attributed to cucurbitacins, which modulate multiple oncogenic signaling cascades. Cucurbitacins are known to inhibit the JAK/STAT3 pathway, suppress PI3K/Akt/mTOR signaling, and induce mitochondrial-mediated apoptosis through caspase-3/9 activation [30,31]. A recent investigation by Kanase et al. [42] demonstrated that wax gourd seed extract exerted anti-proliferative effects against MDA-MB-231 triple-negative breast cancer cells, inducing S-phase cell cycle arrest and modulating intracellular reactive oxygen species (ROS) levels. Additionally, Lee et al. [36] previously reported that the seed extract inhibits basic fibroblast growth factor (bFGF)-induced endothelial cell proliferation and tube formation, indicating anti-angiogenic activity-a critical mechanism for tumor suppression. These findings collectively suggest that wax gourd targets multiple hallmarks of cancer, including sustained proliferative signaling, evasion of apoptosis, and angiogenesis activation [30].

### 4.5. Antimicrobial and Anthelmintic Properties

Antimicrobial investigations of wax gourd have yielded pathogen-dependent results. Natarajan et al. [7] reported that methanolic fruit extracts exhibited antifungal activity against *Candida albicans* (30 mg/mL, concentration-dependent inhibition zones), but failed to demonstrate antibacterial activity against six tested bacterial pathogens including *Staphylococcus aureus* and *Escherichia coli* using the cup plate assay. However, other studies have reported antibacterial effects from seed extracts [43,44], suggesting that antimicrobial activity is influenced by both plant part and extraction methodology.

Collectively, wax gourd demonstrates primarily antifungal activity against *C. albicans*, while antibacterial effects remain inconclusive; its anthelmintic properties against intestinal parasites are supported by in vivo evidence, consistent with traditional applications.

### 4.6. Anti-Diabetic and Hypotensive Actions

Methanol extracts of wax gourd stems demonstrated a progressive reduction in glycemic parameters when administered to alloxan-induced diabetic rodent models. Experimental models with chemically induced diabetes showed concentration-dependent improvement in dyslipidemia patterns following treatment with chloroform-based fruit extracts. Significant suppression of *α*-amylase enzyme activity was observed in peel preparations extracted using methanol, ethanol, and aqueous solvents. Investigations in murine diabetic subjects revealed that ethanol and ethyl acetate leaf derivatives exhibited dose-responsive hypoglycemic properties, particularly in glucose regulation. Fruit-derived methanol extracts displayed appetite-modulating effects through decreased dietary consumption patterns in test animals. Mechanistic studies on aqueous fruit extract’s hexane fraction revealed anti-adipogenic potential via suppression of key adipogenic regulators (leptin, PPARγ, and C/EBPα), leading to diminished lipid storage, elevated glycerol secretion, and altered triglyceride dynamics in 3T3-L1 preadipocyte cultures [5].

Lim investigated the antioxidant effects of wax gourd’s aqueous, chloroform, and butanol extracts in streptozotocin-induced diabetic rats. The chloroform and butanol fractions enhanced hepatic glutathione peroxidase activity when using hydrogen peroxide as substrate, whereas the aqueous fraction reduced both cytosolic glutathione peroxidase and hepatic superoxide dismutase activities. These findings highlight the potential therapeutic application of these extracts in mitigating diabetic complications and lipid peroxidation-induced cellular damage [45].

Through in vivo experiments, in vitro analyses, and cell culture investigations, Nakashima et al. [22] explored the blood pressure-lowering properties of wax gourd juice. Their findings revealed a clear dose–response relationship in blood pressure reduction. Administration of 0.8 mL/kg juice reduced blood pressure by 11 ± 6 mmHg from a baseline of 120 ± 6 mmHg, whereas doubling the dose to 1.6 mL/kg produced a further reduction of 9 ± 7 mmHg. In isolated aortic ring preparations, researchers observed that 1 μL of juice in 5 mL organ baths failed to modify noradrenaline-induced contractions (1 μmol/L). However, escalating concentrations from 3 to 100 μL progressively relaxed the vascular tissue, demonstrating concentration-dependent vasodilation. Comparative analysis showed comparable maximum relaxation effects between wax gourd juice (85.0% ± 3.6%) and acetylcholine (77.9% ± 5.4%), with no statistical difference observed. Subsequent cellular experiments identified nitric oxide (NO) generation as the underlying mechanism responsible for these blood pressure-lowering effects.

Importantly, the anti-diabetic potential of wax gourd has recently been evaluated in a human clinical trial. Che Mohd Zin et al. [46] conducted a placebo-controlled study in patients with type 2 diabetes mellitus, demonstrating that daily consumption of a wax gourd powdered drink significantly improved glycemic control markers compared to placebo. This clinical evidence provides translational support for the traditional use of wax gourd in managing metabolic disorders and underscores the need for further large-scale clinical investigations with standardized preparations. This human trial only enrolled small sample size participants with short intervention period (8 weeks); long-term intervention trials with standardized extract dosage are still required to validate clinical hypoglycemic efficacy.

The anti-diabetic mechanisms of wax gourd are likely multifactorial, involving both pancreatic and extra-pancreatic pathways. Flavonoid constituents such as catechin and quercetin derivatives have been shown in other plant systems to activate AMP-activated protein kinase (AMPK), enhancing GLUT4 translocation and glucose uptake in skeletal muscle, while simultaneously inhibiting hepatic gluconeogenesis through downregulation of PEPCK and G6Pase expression [28]. The high dietary fiber content of wax gourd fruit, particularly the pectic polysaccharides, may further contribute to glycemic regulation by delaying gastric emptying and reducing postprandial glucose absorption [33]. These complementary mechanisms-direct cellular signaling modulation and gastrointestinal effects-provide a mechanistic basis for the observed hypoglycemic activity in both acute and sub-chronic animal models, and warrant further investigation in wax gourd specifically.

### 4.7. Neuroprotective, Antiepileptic, and Antidepressant Actions

Emerging research indicates that wax gourd possesses neuroprotective effects with potential for managing diverse neurological conditions, including neurodegenerative disorders (e.g., Alzheimer’s disease) and seizure disorders. The nootropic potential of wax gourd, known in Ayurveda as Kushmanda, has been systematically reviewed by Neeralakeri et al. [47], who synthesized evidence from pharmacological features, chemical constituents, therapeutic effects, and preclinical investigations to support its classification as a Medhya (cognitive-enhancing) agent. Preclinical investigations have shown that both fresh juice and water-based extracts effectively inhibit seizure episodes, potentially through regulating neural signaling pathways and alleviating oxidative damage, alongside demonstrating marked antidepressant actions in experimental behavioral assessments [48].

Dhingra et al. investigated the antidepressant properties of wax gourd fruit extracts in murine models, suggesting potential interactions with monoaminergic pathways and GABAergic neurotransmission. Their findings demonstrated that administration of 100 mg/kg methanol-based extract substantially reduced immobility durations during behavioral assessments (tail suspension and forced swim tests), mirroring the therapeutic effects observed with conventional antidepressants like imipramine, fluoxetine, and phenelzine. Notably, the treatment exhibited no significant alterations in murine locomotor activity (637.00 ± 15.92 s) compared to control groups (666.17 ± 14.33 s). Researchers proposed that this antidepressant mechanism might involve neuromodulation through enhanced monoamine neurotransmitter levels (norepinephrine, dopamine, serotonin) or suppression of GABAergic signaling pathways [49].

Chandre et al. conducted a clinical investigation involving 35 patients diagnosed with depressive disorders using DSM-IV criteria to evaluate Kushmanda ghrita’s therapeutic effects. Their research demonstrated statistically significant improvements across multiple psychometric measures, including the Hamilton Depression Rating Scale and Hamilton Anxiety Rating Scale, along with both direct and indirect immediate memory assessments, which indicated substantial clinical benefits for depression management [50].

Kumar and Ramu’s comparative analysis revealed that wax gourd fruit juice exhibited seizure-inhibiting properties when tested using the maximal electroshock seizure model. Their findings indicated that administration of 0.9 mL/200 g body weight of fruit juice offered significantly stronger protective effects against convulsions than the aqueous extract at 100 mg/200 g body weight, and both preparations exerted detectable anticonvulsant activity [51].

Nimbal et al. investigated the anxiolytic potential of orally administered ethanolic extract from wax gourd through elevated plus maze (EPM) and light-dark transition (LDT) assessments, concurrently monitoring spontaneous locomotor activity via actophotometry. The experimental treatment demonstrated enhanced duration and frequency of open arm exploration in EPM evaluations. Furthermore, significant improvements were observed in LDT parameters including entry latency, illumination zone duration, and transition frequency between compartments. Notably, the botanical preparation failed to alter spontaneous movement patterns even when administered at elevated concentrations, as quantified through actophotometric analysis [52].

Nimbal et al. assessed the anxiolytic potential of alcoholic extracts through multiple experimental paradigms including open field exploration (OFT), hole board apparatus evaluation, and mirror chamber behavioral analysis in murine subjects. Findings indicated elevated durations in central zone occupancy during OFT measurements following medium and high dosage administration of the alcoholic extract. The hole board model revealed dose-dependent pharmacological effects after seven-day oral treatment, demonstrating prolonged latency until initial head dip responses; concurrent increases in both dipping frequency and cumulative dipping duration were observed. Mirror chamber investigations exhibited marked reductions in entry latency alongside enhanced exploratory behaviors, with treated groups displaying significantly greater chamber entries and prolonged occupancy durations relative to control cohorts receiving vehicle solutions [52].

Ahir et al. conducted a clinical trial assessing the therapeutic efficacy of Kushmandadi Ghrita (containing 16 parts wax gourd fruit extract, 0.25 parts *Glycyrrhiza glabra* root, and 1 part clarified butter) involving 60 participants diagnosed with generalized anxiety disorder. Researchers utilized pre- and post-treatment evaluations of core anxiety indicators as primary assessment criteria, employing sugar-filled capsules as a comparative placebo control. Psychological evaluation metrics incorporated both the Brief Psychiatric Rating Scale and Hamilton Anxiety Rating Scale for comprehensive mental health analysis. The study revealed that this Ayurvedic formulation enhances physiological vitality while calming mental activity, subsequently benefiting sensory perception, cognitive functions, and intellectual capacity. These findings position the preparation as an eco-friendly, non-toxic, and economically viable therapeutic option for managing anxiety disorders through traditional medicinal principles [53].

**Figure 2:**
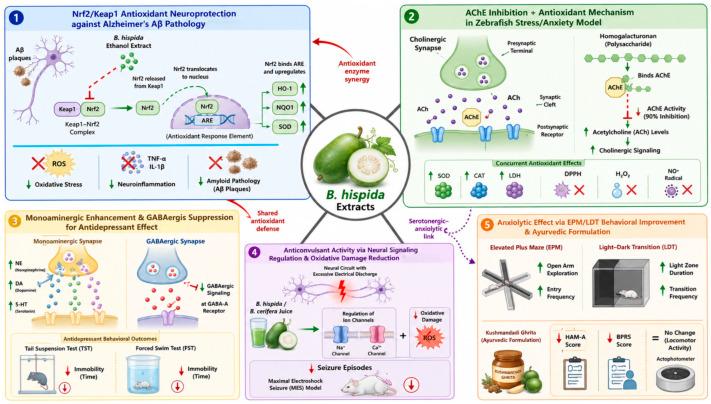
Neuropharmacological mechanisms of wax gourd extracts. Five key actions are illustrated: (**1**) Nrf2/Keap1-mediated neuroprotection against amyloid-*β*-induced pathology (Rapaka et al., 2021 [54]); (**2**) acetylcholinesterase (AChE) inhibition and antioxidant enhancement (Rapaka et al., 2021 [54]); (**3**) Antidepressant effects via monoaminergic/GABAergic modulation (Dhingra & Joshi, 2012 [49]); (**4**) Anticonvulsant activity through neural and oxidative regulation (Chandan et al., 2008 [48]; Kumar & Ramu, 2004 [51]); (**5**) Anxiolytic effects validated by behavioral tests and clinical formulation (Nimbal et al., 2011 [52]; Ahir et al., 2011 [53]). However, this study had a limited sample size (n = 60) and lacked long-term follow-up.

Recent mechanistic studies have provided deeper insights into the neuroprotective pathways activated by wax gourd. Rapaka et al. [54] demonstrated that wax gourd ethanol extract alleviated amyloid-*β* pathology in an Alzheimer’s disease rat model through inhibition of the Keap1/Nrf2 axis, resulting in reduced oxidative stress and neuroinflammation. This Nrf2-mediated antioxidant response represents a key neuroprotective mechanism, as Nrf2 translocation to the nucleus activates cytoprotective genes including HO-1, NQO1, and SOD. Furthermore, Lakshmanagowda et al. [55] reported that wax gourd extract exhibited acetylcholinesterase (AChE) inhibitory activity and modulated antioxidant enzymes (SOD, CAT, LDH) in a zebrafish stress and anxiety model, with molecular docking studies confirming homogalacturonan binding to the AChE receptor. These findings align with the growing evidence that plant-derived polyphenols exert neuroprotection through dual modulation of cholinergic function and oxidative stress pathways [35]. Figure 2 illustrates the neuropharmacological mechanisms of wax gourd extracts, including five core pathways: Nrf2/Keap1-mediated neuroprotection, which is activated via modification of cysteine residues (e.g., Cys151) on Keap1; acetylcholinesterase inhibition; monoaminergic/GABAergic modulation; anticonvulsant activity; and anxiolytic effects validated by behavioral models and clinical evaluation.

### 4.8. Cardiovascular and Diuretic Implications

Jayasree et al. evaluated the diuretic potential of chloroform-extracted Fuzzy melon rind (pericarp) through comparative analysis with normal saline (control) and hydrochlorothiazide (standard). Maintaining consistent fluid volume, experimental groups received 25 mL/kg of 0.9% saline solution, 2.5 mg/kg hydrochlorothiazide, and 100 mg/kg plant extract. Following established methodology, urinary output was monitored over a five-hour period with measurements of total volume, pH levels, and electrolyte excretion patterns for sodium, potassium, and chloride ions. Analysis revealed statistically significant changes including marked elevation in diuresis (114% volume increase), sodium (15%) and chloride (9%) elimination, coupled with reduction (9%) in potassium excretion. These findings demonstrate the extract’s potassium-conserving diuretic properties, validating its traditional applications in edema management and antihypertensive effects through enhanced diuresis [56,57].

The cardiovascular benefits of wax gourd extend beyond its diuretic properties. Nakashima et al. [22] demonstrated that wax gourd juice exerts nitric oxide (NO)-dependent hypotensive effects, mediated through endothelial NO synthase (eNOS) activation and subsequent cyclic guanosine monophosphate (cGMP) signaling in vascular smooth muscle cells. This NO/cGMP pathway is a well-established vasodilatory mechanism, and its activation by wax gourd constituents suggests potential utility in managing essential hypertension. Moon et al. [18] further reported that wax gourd extract attenuated high glucose-induced vascular inflammation in human umbilical vein endothelial cells (HU inhibition-a finding with significant implications for diabetic vascular complications. The ACE-inhibitory activity of wax gourd seed proteins reported by Huang et al. [58] provides an additional renin-angiotensin system-targeted mechanism. Collectively, these findings indicate that wax gourd modulates cardiovascular function through multiple complementary pathways: endothelium-dependent vasodilation, anti-inflammatory vascular protection, and renin-angiotensin system regulation, suppressing ICAM-1 and VCAM-1 expression through *NF-κB*.

### 4.9. Nephroprotective and Hepatoprotective Observations

Studies demonstrate that wax gourd exerts protective effects against drug-induced nephrotoxicity, particularly in cases of acetaminophen-induced renal impairment. These protective effects are attributed to bioactive compounds that attenuate oxidative stress and modulate inflammatory responses, thereby preserving renal structural integrity [59]. Parallel research outcomes reveal the plant’s liver-protective capabilities, with multiple studies documenting how its extracts effectively mitigate hepatic injury caused by toxins, highlighting its therapeutic relevance for metabolic dysfunction and chemically induced liver injury.

The nephroprotective and hepatoprotective activities of wax gourd are an area of growing pharmacological interest, particularly given the global burden of drug-induced organ toxicity. The mechanistic basis for these protective effects is thought to involve the attenuation of oxidative stress-mediated tissue damage, as many nephrotoxic and hepatotoxic agents exert their harmful effects through the generation of reactive oxygen species and subsequent lipid peroxidation. The antioxidant constituents of wax gourd-particularly flavonoids and phenolic acids-may therefore serve as endogenous cytoprotective agents by scavenging free radicals, restoring glutathione homeostasis, and inhibiting the mitochondrial permeability transition pore opening [3].

Bhalodia et al. [60] investigated the renoprotective effects of the methanolic extract of *Benincasa cerifera* fruit against renal ischemia/reperfusion injury in rodent models. Their findings indicated that the extract effectively mitigated renal dysfunction while substantially preserving superoxide dismutase, catalase, and glutathione activities; the improvements in these antioxidant enzyme levels reached statistical significance for superoxide dismutase, showed a marginal trend toward significance for catalase, and were statistically significant for glutathione at the 500 mg/kg dosage relative to control groups. Notably, pretreatment with the extract yielded a highly significant reduction in malondialdehyde concentrations. These nephroprotective properties are attributed primarily to the fruit’s antioxidant capacity in counteracting excessive ROS production during renal oxidative stress.

Roy et al. [61] investigated the protective effect of the aqueous extract of wax gourd fruit against nimesulide-induced hepatotoxicity in an albino rat model. Rats were pretreated with the extract for 14 days before nimesulide administration, and hepatic damage was assessed via serum biochemistry, oxidative stress markers, and histopathological examination. Results showed that wax gourd pretreatment significantly reduced serum levels of SGOT, SGPT, and ALP, elevated hepatic antioxidant enzymes including SOD, CAT, and GSH, and suppressed lipid peroxidation, thereby alleviating hepatocellular degeneration, necrosis, and inflammatory infiltration. The hepatoprotective activity was comparable to that of the reference drug L-ornithine L-aspartate and was mainly mediated through antioxidant, free radical scavenging, and membrane stabilizing mechanisms. These findings support the potential of wax gourd as a safe, natural, and cost-effective agent for mitigating nimesulide-induced liver injury.

Varghese et al. [62] evaluated the nephroprotective efficacy of the hydroalcoholic whole fruit extract of wax gourd against gentamicin-induced renal injury in albino Wistar rats. Rats were assigned to four groups: normal control, gentamicin-treated (80 mg/kg, i.p.), and gentamicin plus HABH at 200 and 400 mg/kg orally, respectively. Nephroprotective effects were evaluated via body and kidney weight, urine output, serum and urinary biochemical indices, renal histopathology, and tissue antioxidant status including Glutathione (GSH) and lipid peroxidation. Administration of HABH significantly attenuated gentamicin-induced increases in urinary electrolytes, urinary glucose, blood urea and creatinine, while improving relative body weight, urinary creatinine excretion and renal glutathione levels. Histopathological examination further revealed that HABH preserved near-normal renal morphology comparable to the control group. Collectively, these findings indicate that the hydroalcoholic fruit extract of wax gourd exerts marked nephroprotective effects against gentamicin-induced kidney damage, likely through maintaining biochemical homeostasis and enhancing renal antioxidant defenses. A comprehensive summary of all documented pharmacological activities of wax gourd, including detailed information on extract types, dosages, experimental models, key effects and underlying molecular mechanisms, is provided in Supplementary Table S2.

### 4.10. Other Potential Biological Activities and Mechanistic Insights

In addition to the biological activities outlined above, wax gourd exhibits several other notable functional properties. Lee et al. [36] identified a potential angiogenic inhibitor in wax gourd seeds, which demonstrates inhibitory effects against both tumor growth and obesity. Furthermore, a study conducted by Qadrie et al. [32] has confirmed that wax gourd seeds possess potent anti-nociceptive and anti-pyretic activities, indicating their potential application in the management of fever and pain. Beyond these findings, Morton reported that wax gourd seeds and seed oil have traditionally been used for tapeworm expulsion, while the wax gourd derived from the fruit is commonly applied topically to alleviate pain associated with wounds.

The methanol extract derived from wax gourd fruit demonstrated promising antidiarrheal effects in rodent models with castor oil-triggered diarrhea. This preparation effectively suppressed prostaglandin E2 (PGE2) synthesis, minimized intestinal fluid accumulation, and decelerated digestive tract movement in subjects receiving activated charcoal meals. At oral dosages of 200, 400, and 600 mg/kg, the extract exhibited dose-dependent inhibition of diarrhea induction, PGE2 elevation, and intestinal fluid retention across three distinct experimental paradigms: castor oil challenge, charcoal transit assessment, and intestinal secretion models [63].

The antidiarrheal mechanism likely involves inhibition of intestinal motility and reduction in fluid secretion, consistent with the traditional use of wax gourd in managing gastrointestinal disturbances. The high pectin content may contribute to stool bulk formation and water binding, while flavonoid constituents could modulate intestinal chloride channels and electrolyte transport [33].

Sabale et al. [64] evaluated a 5% wax gourd fruit extract topical formulation in an ex vivo human skin model using dansyl chloride fluorescence analysis. The extract-treated skin achieved complete fluorescence clearance in 11 days compared to 15–17 days in controls, indicating a highly significant acceleration of epidermal renewal. These findings suggest potential applications of wax gourd extracts in anti-aging cosmeceuticals. 

The anti-aging efficacy of 5% wax gourd extract cream is verified on cadaver skin models; human facial clinical trials remain insufficient to support cosmetic product labeling claims.

**Figure 3:**
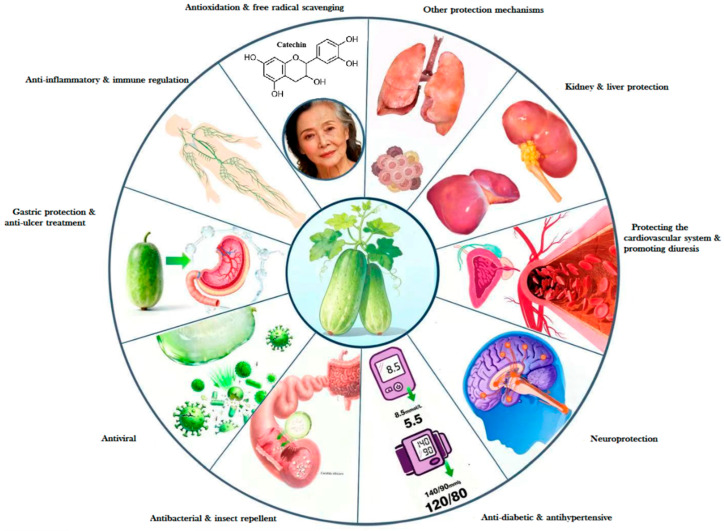
Summary of pharmacological activities of wax gourd.

Figure 3 provides a comprehensive schematic overview of the pharmacological activities of wax gourd documented in preclinical and clinical studies. The diagram illustrates the diverse therapeutic effects-ranging from antioxidant and anti-inflammatory actions to neuroprotective and cardioprotective properties-mediated by the plant’s bioactive constituents through interconnected molecular mechanisms involving oxidative stress modulation, inflammatory pathway suppression, and cellular signaling regulation. A detailed breakdown of these activities by plant part is presented in Table 2.

## 5. Nutraceutical and Functional Food Applications

The documented pharmacological activities provide a scientific foundation for the nutraceutical and functional food applications of wax gourd. The convergence of traditional medicinal use with modern pharmacological evidence underscores the translational potential of wax gourd bioactive compounds in food-based health interventions. The following section examines how these bioactivities have been harnessed in commercial and industrial contexts.

Nutraceuticals—food-derived products providing health benefits beyond basic nutrition—have gained substantial momentum in both scientific research and consumer markets. Vegetable crops of the Cucurbitaceae family, including wax gourd, are recognized as rich sources of bioactive compounds with such potential [3,68]. However, the development of wax gourd-based functional foods requires careful consideration of processing effects, as thermal treatments and storage conditions can significantly alter the phenolic and flavonoid content [17].

### 5.1. Functional Food Products

Wax gourd is widely utilized in the functional food industry across Asia, particularly in traditional preparations. The most commercially significant product is Petha, a traditional candied sweet manufactured from the fleshy mesocarp, which is widely consumed in the Indian subcontinent as both a confectionery item and a digestive aid [5,44,69]. Modern processing techniques have expanded Petha production to include fortified variants incorporating saffron, coconut, and nuts, enhancing both nutritional value and consumer appeal. In China and Southeast Asia, wax gourd juice and beverages represent a growing market segment, marketed for their cooling and diuretic properties in traditional medicine frameworks [3,8].

Beyond traditional preparations, wax gourd has been incorporated into novel functional food formats. Dehydrated wax gourd powder serves as a nutritional supplement and food additive, providing a concentrated source of dietary fiber, phenolic compounds, and minerals [45]. Wax gourd-based instant soup mixes and health drinks have been developed, targeting the growing health-conscious consumer demographic in East Asian markets. The high water content (approximately 96.1%) and low caloric density (13 kcal/100 g) make it particularly suitable for weight management and diabetic-friendly food formulations [12,13].

### 5.2. Bioactive Component Extraction and Industrial Applications

The extraction of bioactive components from wax gourd has attracted considerable industrial interest. Seed oil, rich in linoleic acid (67.37%) and tocopherols, has been explored as a nutraceutical oil with antioxidant and cardioprotective properties [13,48,70]. The protein fraction, containing bioactive peptides with ACE-inhibitory and antioxidant activities, represents a potential source of functional ingredients for the food and pharmaceutical industries [19,49]. Polysaccharide extracts from wax gourd fruit have demonstrated immunomodulatory and prebiotic properties, suggesting applications in functional beverage and dietary supplement formulations [26,50].

Industrial applications extend beyond direct food use. The waxy bloom on mature fruits contains long-chain alkanes and fatty alcohols with potential applications in cosmetic and pharmaceutical formulations [51]. The fruit’s high pectin content has been investigated for use as a natural gelling agent and stabilizer in food processing. Additionally, wax gourd peel extracts, rich in phenolic compounds, have shown promise as natural food preservatives due to their antioxidant and antimicrobial properties [13,27].

### 5.3. Health Benefits in Nutraceutical Context

The nutraceutical potential of wax gourd is underpinned by its phytochemical profile and associated health benefits. Regular consumption has been associated with improved glycemic control, supported by both preclinical evidence and preliminary clinical observations [22,23]. The high dietary fiber content (approximately 0.5 g/100 g fresh weight, concentrated to >58% in seeds) contributes to gastrointestinal health and satiety, while phenolic antioxidants provide protection against oxidative stress-related conditions [12,52]. The hypolipidemic effects observed in animal studies, including reduction in total cholesterol and triglyceride levels, further support the cardiovascular health benefits of wax gourd consumption [23,53]. These multifaceted health benefits position wax gourd as a promising candidate for the development of evidence-based nutraceutical products.

In summary, the nutraceutical landscape of wax gourd extends from traditional food preparations to modern functional food formulations and industrial bioactive extraction. The convergence of its favorable nutritional profile, diverse phytochemical composition, and documented health benefits positions wax gourd as a promising candidate for the development of evidence-based nutraceutical products. However, the realization of this potential will require standardized extraction protocols, rigorous quality control measures, and well-designed human intervention studies to validate the health claims associated with wax gourd-derived products [68].

## 6. Toxicological Profile, Modern Findings and Clinical Perspectives

Toxicological studies have consistently confirmed the favorable safety profile of wax gourd. Administration of fresh juice for three months in rodents produced no significant changes in hematological parameters (WBC, RBC, HB, HCT, MCV, MCH), biochemical markers (blood glucose, urea), or behavioral patterns. The methanolic fruit extract showed no mortality in mice, rats, or guinea pigs at doses ≤ 3.0 g/kg [65,67].

Standardized 70% hydroalcoholic fruit pulp extract was orally safe in both sexes of rats, with a 90-day NOAEL of 1000 mg/kg/day; sub-chronic toxicity assessment revealed no significant alterations in food/water consumption, body weight, relative organ weights, hematological and serum biochemical parameters, or histopathological examination of vital organs [66]. Similarly, ethanolic seed extract exhibited no toxicity at oral doses up to 5000 mg/kg in rat models.

These toxicological findings are encouraging for the development of wax gourd-based nutraceutical products; however, it is important to note that the current safety data are derived primarily from acute and sub-chronic rodent studies. Comprehensive chronic toxicity assessments, reproductive toxicity studies, and genotoxicity evaluations according to OECD guidelines remain to be conducted. Furthermore, the safety profile of concentrated bioactive extracts—particularly cucurbitacin-enriched fractions—may differ substantially from that of whole fruit preparations, as cucurbitacins are known to exhibit dose-dependent cytotoxicity, with reported no-observed-adverse-effect levels (NOAELs) in rodent models ranging from 10 to 50 mg/kg body weight for isolated cucurbitacins [30,31]. The establishment of appropriate upper intake limits and standardized quality control measures will be essential for regulatory approval of wax gourd nutraceutical products.

Despite abundant research on wax gourd, the bioavailability and pharmacokinetic characteristics of its bioactive compounds remain poorly characterized. Flavonoids such as those identified in wax gourd (e.g., catechin, naringenin, quercetin derivatives) are known to exhibit poor oral bioavailability due to extensive first-pass metabolism, low aqueous solubility, and rapid elimination [28]. Similarly, cucurbitacins, while potent in cellular models, present significant pharmacokinetic challenges including low oral absorption and dose-limiting toxicity that complicate their clinical translation [31]. The bioactive peptides (*α*- and *β*-benincasins) and ribosome-inactivating proteins (hispin) face additional barriers related to protein stability and gastrointestinal degradation. Recent fermentation-based processing of wax gourd has shown promise in enhancing the bioavailability of certain compounds, notably via *Lactobacillus plantarum* fermentation, which increased phenolic monomer release by 30–40%; Choi et al. [71] identified novel marker compounds including 2-furoic acid, 2,3-dihydroxybenzoic acid, and rubinaphthin A in fermented wax gourd preparations using UPLC-QTOF-MS/MS, suggesting that biotransformation during fermentation may improve the accessibility of bioactive constituents. Future studies should prioritize pharmacokinetic characterization of key wax gourd compounds and evaluate novel delivery strategies such as nanoencapsulation and phospholipid complexes to enhance their therapeutic potential. All available toxicological evidence derives from rodent acute/subchronic trials; human safety assessments and long-term oral toxicity data are still absent.

## 7. Concluding Remarks and Future Perspectives

This review synthesizes the phytochemical, pharmacological, and nutraceutical dimensions of wax gourd (*Benincasa hispida*), a monotypic cucurbit with substantial medicinal and agricultural value. Phytochemical profiling has revealed a diverse array of bioactive constituents—including cucurbitacins, flavonoids, triterpenoids, phenolic acids, sterols, and bioactive peptides—that collectively underpin its broad therapeutic spectrum. Pharmacological studies have substantiated antioxidant, anti-inflammatory, gastroprotective, neuroprotective, antidiabetic, cardioprotective, and nephroprotective activities, mediated through mechanisms such as NF-κB pathway suppression, COX-2 inhibition, oxidative stress modulation, and apoptotic regulation.

Recent genomic advances have begun to elucidate the molecular basis of agronomic traits in wax gourd. The initial 983.84 Mb draft genome assembly (23,241 genes) [72] has been superseded by a chromosome-level reference genome (31,562 genes) reported by Luo et al. [73], providing an enhanced resource for gene discovery and marker-assisted breeding. Key genetic determinants have been identified, including the *BhAPRR2* gene governing peel color variation via a 4-bp coding-region insertion [74], along with multiple QTL loci controlling fruit size, shape, and flesh thickness [73]. Transcriptomic analyses have further clarified disease resistance mechanisms [75] and pericarp color regulation [9], while comparative chloroplast genomics have illuminated evolutionary relationships within *Benincaseae* [76]. Collectively, these resources establish a robust framework for molecular breeding programs targeting improved nutritional quality, stress tolerance, and disease resistance.

Looking forward, six priority areas merit focused investigation: (1) Pharmacokinetic and pharmacodynamic profiling of key bioactive compounds using advanced analytical platforms (LC-MS/MS, PET imaging) to establish dose-exposure-response relationships; (2) Multi-center, randomized controlled trials with standardized extracts to validate efficacy in metabolic syndrome, neurodegenerative disorders, and inflammatory conditions—the three areas with the strongest preclinical evidence; (3) Network pharmacology and systems biology approaches to elucidate compound synergy beyond single-compound paradigms; (4) Genomic-assisted breeding leveraging the chromosome-level assembly and the *BhAPRR2* locus to develop cultivars with optimized bioactive profiles; (5) Advanced delivery systems (nanoencapsulation, phytosomes, co-crystallization) to overcome bioavailability limitations of flavonoids and cucurbitacins; (6) Comprehensive safety assessment following OECD guidelines, including chronic toxicity, genotoxicity, reproductive toxicity, and herb-drug interaction studies.

The translation of preclinical findings to clinical applications remains a critical frontier. While cell-based and animal studies are promising, human clinical evidence is still limited. The recent placebo-controlled trial by Che Mohd Zin et al. [46], demonstrating improved glycemic control in type 2 diabetes patients with a wax gourd powdered drink, represents an important step toward clinical validation. However, large-scale, randomized, double-blind trials with standardized extracts and defined dosage regimens are urgently needed.

The convergence of traditional ethnopharmacological knowledge, contemporary genomic insights, and modern pharmacological evidence positions wax gourd as a model for translational botanical research. Multidisciplinary strategies integrating metabolomics, bioinformatics, molecular breeding, and clinical investigation will be essential to fully realize the health-promoting potential of this remarkable cucurbit. Addressing the gaps in clinical evidence, bioavailability, and safety assessment will be key to positioning wax gourd-derived products within the evidence-based nutraceutical and pharmaceutical landscape.

## Figures and Tables

**Table 1:** Comprehensive nutritional composition of wax gourd (*Benincasa hispida*) fruit (per 100 g edible portion).

Component	Amount	Unit
Moisture	96.1	%
Energy	13	kcal
Dry matter	3.9	g
Protein	0.4	g
Total lipid (fat)	0.2	g
Carbohydrate (by difference)	1.9	g
Fiber, total dietary	0.8	g
Epicuticular wax bloom	0.3	g
Calcium, Ca	30	mg
Iron, Fe	0.8	mg
Magnesium, Mg	10	mg
Phosphorus, P	19	mg
Potassium, K	111	mg
Sodium, Na	6	mg
Zinc, Zn	0.61	mg
Copper, Cu	0.023	mg
Manganese, Mn	0.058	mg
Selenium, Se	0.2	µg
Vitamin C (total ascorbic acid)	13	mg
Thiamine (B1)	0.04	mg
Riboflavin (B2)	0.11	mg
Niacin (B3)	0.4	mg
Pantothenic acid (B5)	0.133	mg
Vitamin B6	0.035	mg
Folate, total	5	µg
Lysine	0.009	g
Methionine	0.003	g

**Table 2:** Medicinal and pharmacological properties of different parts of wax gourd.

Part	Medicinal and Pharmacological Properties	References
Pulp	Anti-inflammatory, anti-ulcer, anti-depressant, anti-histaminic, antioxidant, anti-compulsive, anti-diarrheal and anti-obesity activities; beneficial effects in allergic inflammation, insanity and epilepsy; preventive and curative effects in nervous disorder, intestinal worms, jaundice, diabetes, leucorrhoea, stomach and bile problems; potential uses as diuretic, laxative, aphrodisiac, clearing heat and detoxificant; used for Alzheimer’s disease treatment, facial eruption, inhibition of angiotensin converting enzyme (ACE); nootropic effects.	[20,63]
Seed	Anti-angiogenic, anti-tumor, antioxidant, anti-nociceptive, and anti-pyretic activities; soporific potential, and beneficial effects for the brain and liver; used for the treatment of syphilis, cardiovascular diseases, inhibition of angiotensin converting enzyme (ACE), expel intestinal worm and softening or soothing the skin.	[10,11,32,58,65]
Peel	Antioxidant activity; inhibition of angiotensin converting enzyme (ACE), Antioxidant, antimicrobial, food preservation.	[13,27,66,67]
Leaf	Anti-inflammatory, antimicrobial	[3,44]
Root	Anti-inflammatory, cytotoxic	[16,58]

## Data Availability

The raw data supporting the conclusions of this manuscript will be made available by the authors, without undue reservation, to any qualified researchers.

## Supplementary Materials

The following supporting information can be downloaded at: https://www.mdpi.com/article/10.3390/plants15132020/s1, Figure S1. Chemical structures of representative bioactive compounds identified in various tissues of. (a) Hispidin, a unique polyphenolic compound with documented antioxidant, anti-inflammatory and neuroprotective properties, predominantly found in the fruit flesh. (b) Flavonoids and phenolic acids, the primary antioxidant constituents of wax gourd, which are most abundant in the peel and leaves. (c) Tocopherols/tocotrienols (vitamin E derivatives), signature cucurbitane-type triterpenoids (cucurbitacin B and D), and phytosterols. These compounds mediate the anticancer, anti-inflammatory, lipid-regulating and anti-diabetic, anti-cancer, and multi-organ protective effects (e.g., nephro-, hepato-, and cardio-protective) of cucurbitacins, with cucurbitacins being particularly enriched in the fruit peel. Supplementary Table S1. Chemical constituents found in whole plant of wax gourd [10,12,13,14,15,16,23]. Table S2. Comprehensive summary of pharmacological activities of wax gourd.

## Abbreviations

The following abbreviations are used in this manuscript:

AChE	Acetylcholinesterase
ACE	Angiotensin-Converting Enzyme
AMPK	AMP-activated Protein Kinase
A*β*	Amyloid-*β*
BSM	Benincasa Seed Mitogen
CAT	Catalase
COX	Cyclooxygenase
DPPH	2,2-Diphenyl-1-picrylhydrazyl
eNOS	Endothelial Nitric Oxide Synthase
EMT	Epithelial–Mesenchymal Transition
GABA	*γ*-Aminobutyric Acid
GAE	Gallic Acid Equivalent
GSH	Glutathione
HO-1	Heme Oxygenase-1
IC_50_	Half Maximal Inhibitory Concentration
iNOS	Inducible Nitric Oxide Synthase
IL	Interleukin
JAK	Janus Kinase
LPS	Lipopolysaccharide
LDH	Lactate Dehydrogenase
MDA	Malondialdehyde
mTOR	Mammalian Target of Rapamycin
NF-*κ*B	Nuclear Factor Kappa-B
NO	Nitric Oxide
NOAEL	No Observed Adverse Effect Level
NQO1	NAD(P)H:Quinone Oxidoreductase 1
Nrf2	Nuclear Factor Erythroid 2-Related Factor 2
PI3K	Phosphatidylinositol 3-Kinase
PGE_2_	Prostaglandin E₂
PPARγ	Peroxisome Proliferator-Activated Receptor γ
QTL	Quantitative Trait Locus
ROS	Reactive Oxygen Species
RIP	Ribosome-Inactivating Protein
SAR	Structure-Activity Relationship
SOD	Superoxide Dismutase
STAT3	Signal Transducer and Activator of Transcription 3
TLR4	Toll-like Receptor 4
TNF-α	Tumor Necrosis Factor-α
TPC	Total Phenolic Content
WGP	Wax Gourd Polysaccharide
DW	Dry Weight
HAT	Hydrogen Atom Transfer
SET	Single Electron Transfer
cGMP	Cyclic Guanosine Monophosphate
HUVEC	Human Umbilical Vein Endothelial Cell
ICAM-1	Intercellular Adhesion Molecule-1
VCAM-1	Vascular Cell Adhesion Molecule-1
SGOT	Serum Glutamic Oxaloacetic Transaminase
SGPT	Serum Glutamic Pyruvic Transaminase
ALP	Alkaline Phosphatase
UPLC-QTOF-MS/MS	Ultra-Performance Liquid Chromatography-Quadrupole Time-of-Flight Tandem Mass Spectrometry
SNP	Single Nucleotide Polymorphism
