# Supplementary material for: Medicinal Ingredients of Wax Gourd (Benincasa hispida (Thunb.) Cogn.): An Integrated Review of Phytochemistry, Pharmacology, and Nutraceutical Applications
Source: Plants (Basel). 2026 Jun 30;15(13):2020. doi: 10.3390/plants15132020 (PMC13363735; doi:10.3390/plants15132020)
Supplement: Supplementary file 1 [file plants-15-02020-s001.zip › plants-4370194-supplementary.pdf]

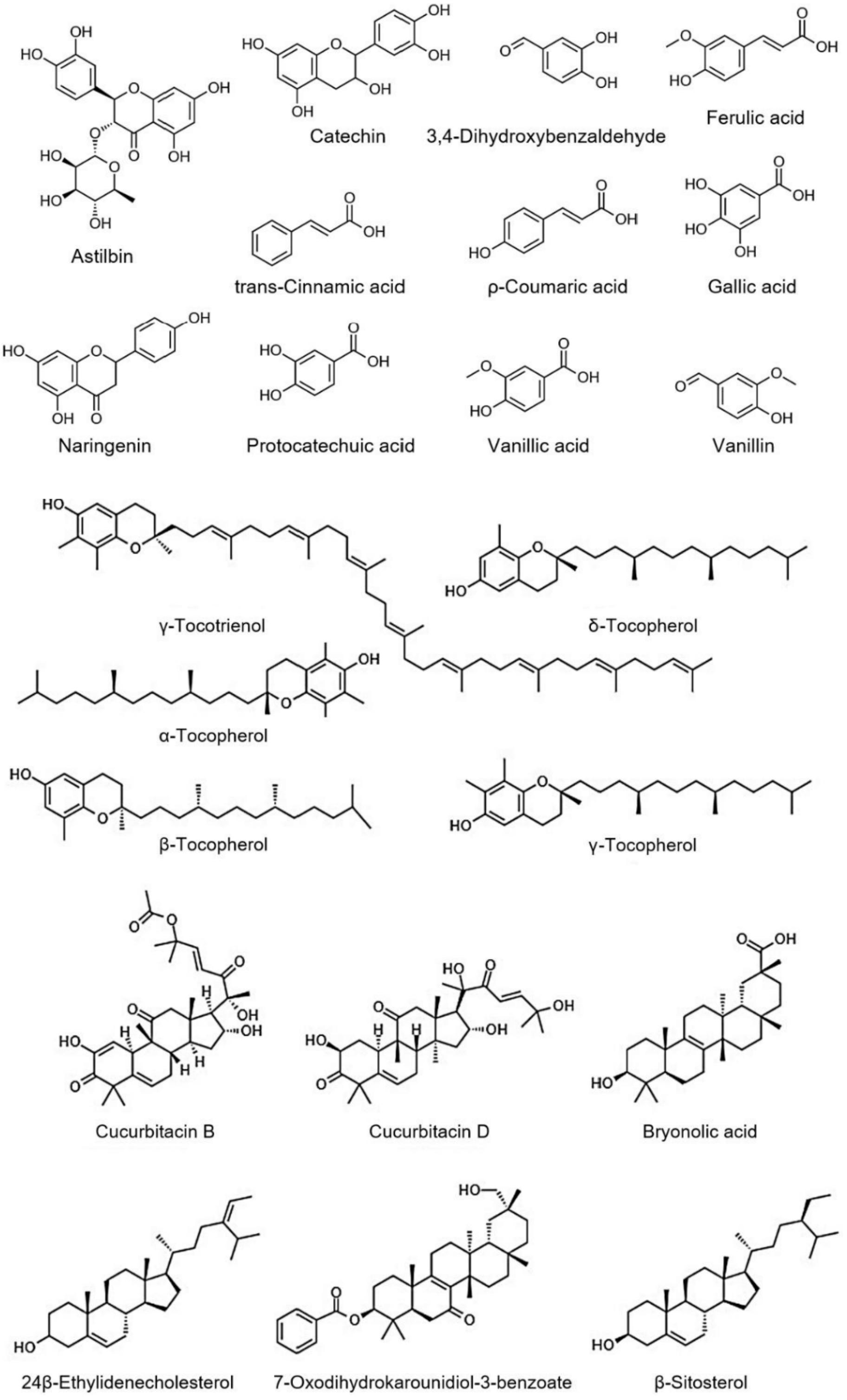

Supplementary Figure S1. Chemical structures of representative bioactive compounds identified in various tissues of *Benincasa hispida*

Supplementary Table S1. Chemical constituents found in whole plant of wax gourd [10,12–16,23].

Part of Plant	Chemical Constituents

Root	Pentacyclic triterpene, Bryonolic acid, 7-oxodihydrokarounidiol-3-benzoate, Alsenusol B, Isomultiflorenol
Fruit	Lupeol, β-sitosterol, cucurbitin, rhamnose, mannitol, triacontenol, triterpenoids, flavonoids, glycosides, saccharides, carotenes, vitamins,Benincasides A-E, Benincasaponins A-I, Bryonolic acid, Multiflorenol, Isomultiflorenol, Alsenusol B,Dietary fiber, Pectin, Ascorbic acid, Thiamine, Riboflavin, Niacin, Carotenoids (β-Carotene)[13]
Fruit (peel)	Cucurbitacin B, Cucurbitacin E, Cucurbitacin D, Lupeol, β-Sitosterol, Campesterol, Stigmasterol
Fruit (flesh)	Cucurbitacin B, Cucurbitacin D, Benincasins A-I, Isorhamnetin, Quercetin, Kaempferol
Fruit (whole)	Rutin, Isovitexin, Catechin, Naringenin, Quercetin, Apigenin, Luteolin, Myricetin, Anthocyanins
Fruit (polysaccharides)	Wax gourd polysaccharide (WGP), BSM (Benincasa Seed Mitogen), Uronic acid-containing heteropolysaccharides
Fruit (volatile)	Hexanal, (E)-2-Nonenal, (E,Z)-2,6-Nonadienal, 2-Undecenal, Methyl salicylate, α-Copaene
Stem	Cucurbitacins, Triterpenoids, Sterols
Seed	24β-ethylidene cholesterol-7-enol, 24-β-ethyl cholesterol, saponin, urea, citrulline, linoleic acid, oleic acid, isomultiflorenol, trigonelline, coffearin, osmotin, beta-sitosterol, stigmast-5-ene-3-beta-ol, 5-methylcytosine, cucurbitacin,α-Benincasin, β-Benincasin, Hispin, Cucurbitin, Trigonelline, Multiogtin, Benincasa agglutinin,Protease inhibitors, Trypsin inhibitors, α-Amylase inhibitors, Ribosome-inactivating proteins[19]
Seed oil	Linoleic acid (67.37%), Palmitic acid (17.11%), Oleic acid (10.21%), γ-Tocopherol, α-Tocopherol, β-Tocopherol
Leaf	Uronic acid, alnusenol, multiflorenol, isomultiflorenol, iso-vitexin, lupeol, lupeol acetate, beta-sitosterol, Rutin, Isovitexin,

	Catechin, Naringenin, Astilbine, Gallic acid, Caffeic acid, Ferulic acid[69]

Supplementary Table S2. Comprehensive summary of pharmacological activities of Benincasa hispida.

Pharmacological Activity	Extract/Compound	Dose/Concentration	Model	Key Effects	Mechanism of Action	References
Anti-angiogenic	Seed protein extract	Varied	HUVEC tube formation assay	Inhibition of angiogenesis	Suppression of VEGF signaling	[36]
Anti-obesity	Seed protein extract	Varied	3T3-L1 adipocyte model	Inhibition of adipogenesis	PPARγ pathway suppression	[36]
Neuroprotective (anti-Alzheimer's)	Ethanolic fruit extract	Varied	Aβ-induced rat model	Alleviated Aβ pathology	Keap1/Nrf2 axis inhibition	[54]
Wound healing	Aqueous fruit extract	5% w/w ointment	Excision wound model (rat)	Accelerated wound closure	Collagen synthesis promotion	[55]
Anti-urolithiatic	Aqueous fruit extract	Varied	Ethylene glycol-induced (rat)	Reduced calcium oxalate crystals	Diuretic and crystallization inhibition	[42]
Cardioprotective	Methanolic fruit extract	Varied	Isoproterenol-induced (rat)	Preserved cardiac markers	Antioxidant, membrane stabilization	[22]
Hypolipidemic	Aqueous fruit extract	Varied	High-fat diet (rat)	Reduced total cholesterol, LDL	HMG-CoA reductase inhibition	[22]
Anti-diarrheal	Methanolic fruit extract	200–400 mg/kg	Castor oil-induced (rat)	Reduced diarrheal frequency	Anti-motility and anti-secretory	[71]
Anti-nociceptive	Ethanolic seed extract	Varied	Acetic acid writhing test (mouse)	Reduced writhing	Peripheral analgesic mechanism	[70]

Anti-pyretic	Ethanolic seed extract	Varied	Brewer's yeast-induced (rat)	Reduced body temperat ure	Hypothalamic prostaglandin modulation	[70]
